# MicroRNA-1246 enhances migration and invasion through CADM1 in hepatocellular carcinoma

**DOI:** 10.1186/1471-2407-14-616

**Published:** 2014-08-27

**Authors:** Zhao Sun, Changting Meng, Shihua Wang, Na Zhou, Mei Guan, Chunmei Bai, Shan Lu, Qin Han, Robert Chunhua Zhao

**Affiliations:** Department of Oncology, Peking Union Medical College Hospital, Chinese Academy of Medical Sciences and Peking Union Medical College, Beijing, People’s Republic of China; Institute of Basic Medical Sciences Chinese Academy of Medical Sciences, School of Basic Medicine Peking Union Medical College, Beijing, People’s Republic of China; China National Center for Biotechnology Development, Beijing, People’s Republic of China; Center of Translational medicine, Peking Union Medical College Hospital, Beijing, People’s Republic of China

**Keywords:** Hepatocellular carcinoma, Invasion, MicroRNA-1246, CADM1

## Abstract

**Background:**

The aberrant expression of microRNAs has been demonstrated to play a crucial role in the initiation and progression of hepatocarcinoma. miR-1246 expression in High invasive ability cell line than significantly higher than that in low invasive ability cell line.

**Methods:**

Transwell chambers (8-uM pore size; Costar) were used in the in vitro migration and invison anssay. Dual luciferase reporter gene construct and Dual luciferase reporter assay to identify the target of miR-1246. CADM1 expression was evaluated by immunohistochemistric staining. The clinical manifestations, treatments and survival were collected for statistical analysis.

**Results:**

Inhibition of miR-1246 effectively reduced migration and invasion of hepatocellular carcinoma cell lines. Bioinformatics and luciferase reporter assay revealed that miR-1246 specifically targeted the 3′-UTR of Cell adhesion molecule 1 and regulated its expression. Down-regulation of CADM1 enhanced migration and invasion of HCC cell lines. Furthermore, in tumor tissues obtained from liver cancer patients, the expression of miR-1246 was negatively correlated with CADM1 and the high expression of miR-1246 combined with low expression of CADM1 might serve as a risk factor for stage1 liver cancer patients.

**Conclusions:**

Our study showed that miR-1246, by down-regulation CADM1, enhances migration and invasion in HCC cells.

**Electronic supplementary material:**

The online version of this article (doi:10.1186/1471-2407-14-616) contains supplementary material, which is available to authorized users.

## Highlights

1. miR-1246 is highly expressed in more metastatic human carcinoma cells.
2. Inhibition of miR-1246 effectively reduced migration and invasion of hepatocellular carcinoma (HCC) cell lines.
3. CADM1 is a direct target of miR-1246.
4. The expression of miR-1246 was negatively correlated with CADM1.
5. The high expression of miR-1246 combined with low expression of CADM1 might serve as a risk factor for stage1 liver cancer patients.

## Background

Liver cancer in men is the fifth most frequently diagnosed cancer worldwide but the second most common cause of cancer death. In women, it is the seventh most frequently diagnosed cancer and the sixth leading cause of cancer death. The regions of high incidence include Eastern and South-eastern Asia, as well as Middle and Western Africa. Approximately 700,000 new liver cancer cases are diagnosed worldwide every year, and half of which are from China [1, 2]. Among primary liver cancers, hepatocellular carcinoma (HCC) is the major histological subtype. Rapid malignant progression, dismal survival rate and high frequency of recurrence and metastasis remain the crux of HCC treatment. Investigation of mechanisms involved in HCC recurrence and metastasis might lead to development of novel therapeutic strategies.

MicroRNAs (miRNAs) are small single-stranded RNA molecules, which have emerged as important post-transcriptional regulators of gene expression. Many miRNAs are differentially expressed between malignant and normal tissues and they might function as either oncogenes or tumor suppressors. Through regulating the expression of proteins involved in signaling pathways, miRNAs play an important role in various biological processes associated with cancer, such as cell proliferation, differentiation and invasion [3–6].

Currently, a few studies have revealed the function of miRNAs in the development and progression of HCC [7]. For example, miR-199a-5p was reported be down-regulated in HCC tissues, which contributes to increased cell invasion by functional deregulation of DDR1 [8]. Wu et al. found overexpression of miR-142-3p decreased RAC1 protein levels and subsequently suppressed migration and invasion of HCC cell lines (SMMC7721and QGY7703) [9]. Bae et al. reported that miR-29c could function as a tumor suppressor whose loss or suppression caused aberrant SIRT1 overexpression and promoted liver tumorigenesis [10]. These studies bring insight into the roles of aberrantly expressed miRNAs in HCC, which may help identify biomarkers for patients with a higher probability of metastasis.

Hep11 and Hep12 are two cell lines established from primary tumor and recurrent tumor respectively of the same patient [11, 12]. They possess significantly different proliferative and invasive ability. Therefore, we analyzed the differential expression of miRNAs between Hep11 and Hep12 using microRNA microarray [13] and discovered extraordinarily high expression levels of miR-1246 in both cell lines, with miR-1246 expression in Hep12 significantly higher than that in Hep11. Interestingly, qRT-PCR showed that expression levels of miR-1246 were even higher than the internal reference gene U6. What is the biological significance of such abundant miR-1246 expression in HCC? Here, we showed that microRNA-1246 enhances migration and invasion through CADM1.

## Method

### Tumor cell culture

The human carcinoma cell (HCC) lines HepG2, SMMC7721 and BEL7402 were maintained in DMEM medium (Gibco, Paisley, UK) supplemented with 10% fetal calf serum (FCS, Gibco, Paisley, UK) in humidified 5% CO2/95% atmosphere at 37°C. HepG2 (human hepatic carcinoma), SMMC 7721 (human hepatic carcinoma) and BEL7402 (human hepatic carcinoma) were purchased from Cell Resource Center, IBMS, CAMS/PUMC).

### In vitro Cell proliferation assay

SMMC7721 cells transfected with the miR-1246 mimic, mimic control, miR-1246 inhibitor and inhibitor control were detached with trypsin and seeded in 96-well plates (5 × 10^3^/well). Cell proliferation was detected by CellTiter 96® AQueous (Promega, Madison, WI, USA) every 24 hours.

### miRNA and siRNA transfection

The synthetic miR-1246 mimic (forward, 5′- AAUGGAUUUUUGGAGCAGG - 3′; reverse, 5′- UGCUCCAAAAAUCCAUU TT- 3′), miR-1246 inhibitor (5′- CCUGCUCCAAAAAUCCAUU- 3′), mimic control (forward, 5′-UUCUCCGAACGUGUCACGUTT-3′; reverse, 5′-ACGUGACACGUUCGGAGAATT-3′) and inhibitor control (5′-UUGUACUACACAAAAGUACUG-3′) were purchased from GenePharma (GenePharma Inc., Shanghai, China). The siRNAs for CADM1 was synthesized (Invitrogen Inc.) and the sequence was list in Additional file 1: Table S1. The nucleotide sequences were delivered into human HCC cell lines by Amaxa Nucleofector® following the manufacturer’s instructions. Briefly, cell pellets were collected by 90 × g centrifugation at room temperature for 10 min and resuspended in 100 μl of Nucleofector Solution to a final concentration of 1 × 10^6^ cells/100 μl. Each 100 μl sample was transferred into an Amaxa-certified cuvette and underwent nucleofection using the appropriate Nucleofector program. The program for transfecting HepG2 and SMMC7721 was T028. After nucleofection, each sample was immediately transferred from the cuvette to a culture plate in 2 ml of medium [14].

### RNA reverse transcription and qRT-PCR

Total RNA was extracted using the Trizol total RNA isolation reagent (Invitrogen) and purified with the Column DNA Erasol kit (TIANGEN, Beijing, China) according to the manufacturers’ instructions. mRNA levels were assessed with qRT-PCR using SYBR Green I (TaKaRa, Dalian, China). The gene expression level was normalized to the endogenous reference gene *GAPDH*. The experiments were performed in triplicate. The primers for qRT-PCR are listed in Additional file 1: Table S2. The primers for miR-1246 and U6 were purchased from QIAGEN (Additional file 1: TableS3). Reverse transcription of miRNAs was performed with a miScript Reverse Transcription Kit (QIAGEN, Duesseldorf, Germany). The expression of mature miRNAs was determined using miRNA-specific quantitative qRT-PCR (TaKaRa, Dalian, China). The expression levels were normalized to the U6 endogenous control and measured by the comparative Ct (ΔΔCt) method.

### Western blot analysis

After washing twice with PBS, cells were lysed in ice-cold Radio Immunoprecipitation Assay (RIPA) lysis buffer (Beyotime, Nanjing, China) and manually scraped from culture plates. Proteins were separated on 10% sodium dodecyl sulfate–polyacrylamide gel electrophoresis (SDS-PAGE) gels, electroblotted onto a polyvinylidene difluoride (PVDF) membrane and incubated with anti-CADM1 antibody (1/1000; Santa Cruz Biotechnology, Santa Cruz, CA), anti-GAPDH antibody (1/1000; Santa Cruz Biotechnology, Santa Cruz, CA), followed by incubation with a secondary anti-rabbit or anti-mouse horseradish peroxidase-conjugated antibody (Zhongshan, Beijing, China). Antibody-antigen complexes were detected using a chemiluminescent ECL reagent (Millipore).

### Dual luciferase reporter gene construct and Dual luciferase reporter assay

An 66 bp fragment of the CADM1 3′UTR containing the predicted binding site for hsa-miR-1246 and flanking sequence on each side was synthetized with a short extension containing cleavage sites for XbaI (5′ end) and NotI (3′ end); a second fragment containing a mutated sequence of the binding site was also synthesized. The two constructs were termed WT (CADM1-wild type) and MT (CADM1-mutant). The fragments were cloned into the pRL-TK vector (Promega Corporation, Madison, WI) downstream of the renilla luciferase reporter gene. The sequence of the miR-1246 binding site and mutant site are shown in Additional file 1: Table S4. Each vector, along with 100 ng of pGL3 and 200 nmol/L miR-1246 mimic or mimic control, was transfected into 293 T cells using Lipofectamine 2000 (Invitrogen, Carlsbad, CA) according to the manufacturer’s instructions. Cells were harvested 24 hours after transfection and assayed for renilla and firefly luciferase activity using the Dual-Luciferase Reporter Assay System (Promega, Madison, WI, USA).

### In vitro migration and invasion assay

Transwell chambers (8-uM pore size; Costar) were used in the in vitro migration assay. HCC cells were transfected with the miR-1246 mimic, mimic control, miR-1246 inhibitor, inhibitor control, Si-CADM1 and SiRNA control. After 48 hours, cells were detached with trypsin, washed with PBS and resuspended in serum-free medium. Two hundred microliters of cell suspension (1 × 10^6^ cells/ ml of Hep11, 5 × 10^5^ cells/ ml of SMMC7721 and BEL7402) was added to the upper chamber, and 500 μl of complete medium was added to the bottom well. The cells that had not migrated were removed from the upper surfaces of the filters using cotton swabs, and the cells that had migrated to the lower surfaces of the filters were fixed with 4% Paraformaldehyde solution and stained with Crystal Violet. Images of three random fields (10× magnification) were captured from each membrane, and the number of migratory cells was counted. Similar inserts coated with Matrigel were used to determine the invasive potential.

### Clinical samples

38 paraffin liver cancer tissue samples were obtained from patients with hepatocellular carcinoma who underwent surgery in Peking Union Medical College Hospital from 2009–2010. All the patients were positive for HBV infection and none of them were found to have distant metastases before surgery. Cancer stages were classified according to TNM. They all underwent 1–3 times of transcatheter hepatic arterial chemoembolization (TACE) and the chemotherapy drugs were 5-Fu, epirubicin, HydroxycamptotbecineInjection (HCPT). The basic condition of patients were list in Additional file 1: Table S5.

CADM1 expression was evaluated by immunohistochemistric staining. Briefly, after 5-um sections were deparaffinized, antigen retrieval was performed by use of heat-induced epitoperetrieval with 10 mM citrate buffer. Sections were incubated with a monoclonal antibody against CADM1 (Abcam, UK) at 1 : 250 dilution. The CADM1 antibody was detected using the avidin-biotin-peroxidase technique (DakoLSAB Kit, Dako). The expression levels of CADM1 were determined by a pathologist. The classification of “-, +, ++” was defined by the percentage of CADM1 positive cells at the level of <10%, 10-50%, and 51-100%, respectively.

### Statistical analysis

Comparisons between groups were analyzed using t-tests (two-sided). Differences with P values of less than 0.05 are considered significant. Correlation between miR-1246 and CADM1 expression was determined by SPSS assay and correlate bivariate kendallis tau-b assay. Kaplan-Meier was used to analyze disease-free survival (DFS) of the patients.

## Results

### miR-1246 expression in several HCC cell lines

Hep11 and Hep12 are two cell lines that were previously established by primary culture from the same patient [11, 12]. Hep11 cells were acquired after the patient’s first radical surgery for primary HCC, when no metastasis was found. In contrast, Hep12 cells were acquired from a surgically resected tumor that relapsed after failed chemotherapy and radiotherapy. Our previous study analyzed the differential expression of miRNAs between Hep11 and Hep12 using microRNA microarray [13] and discovered differential expression of miR-1246. Here, using quantitative real-time PCR, we confirmed that expression of miR-1246 in Hep12 is over 700-fold higher than in Hep11 (Additional file 2: Figure S1). We also evaluated miR-1246 expression in other HCC cell lines. Interestingly, miR-1246 expression in HepG2, SMMC-7721, Hep3b and BEL-7402 is 6, 11, 14 and 22-fold higher, respectively, than in Hep11 (Additional file 3: Figure S2).

### miR-1246 promotes migration and invasion of HCC cell lines in vitro

**Figure 1:**
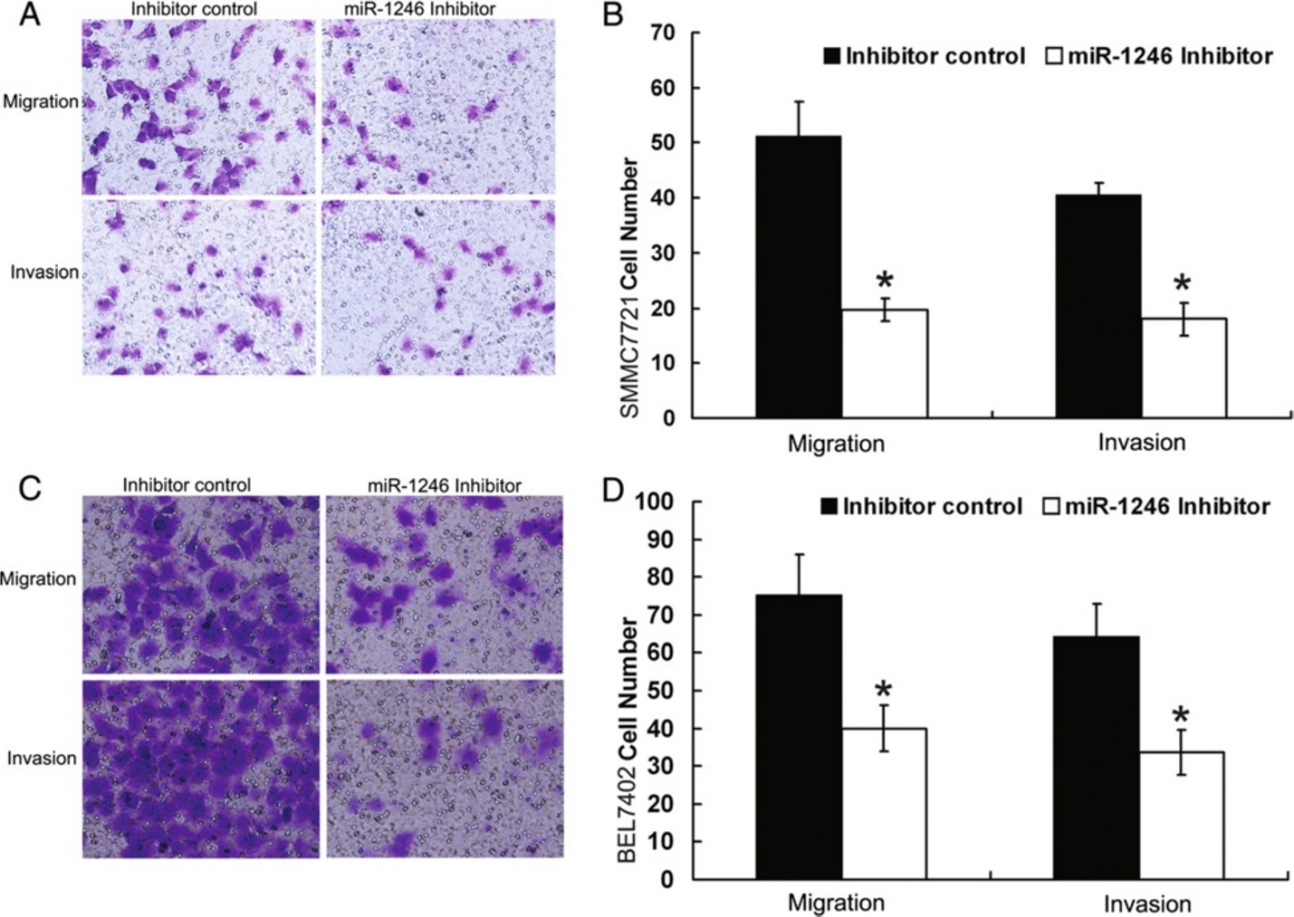
**Inhibition of miR-1246 reduced migration and invasion of SMMC7721 and BEL7402. (A, B)** Transwell migration (n = 4) and invasion (n = 4) assays showed that SMMC7721 cells transfected with the miR-1246 inhibitor (800 nM) had lower invasive and migratory potentials than the control (inhibitor control). **(A)** is a microscopic image of crystal violet staining; **(B)** shows the statistical results. **(C, D)** Transwell migration (n = 4) and invasion (n = 4) assays showed that BEL7402 cells transfected with the miR-1246 inhibitor (800 nM) had lower invasive and migratory potentials than the control (inhibitor control). **(C)** is a microscopic image of crystal violet staining; **(D)** shows the statistical results. Data represent the mean ± SD of four independent experiments. *P < 0.01.

**Figure 2:**
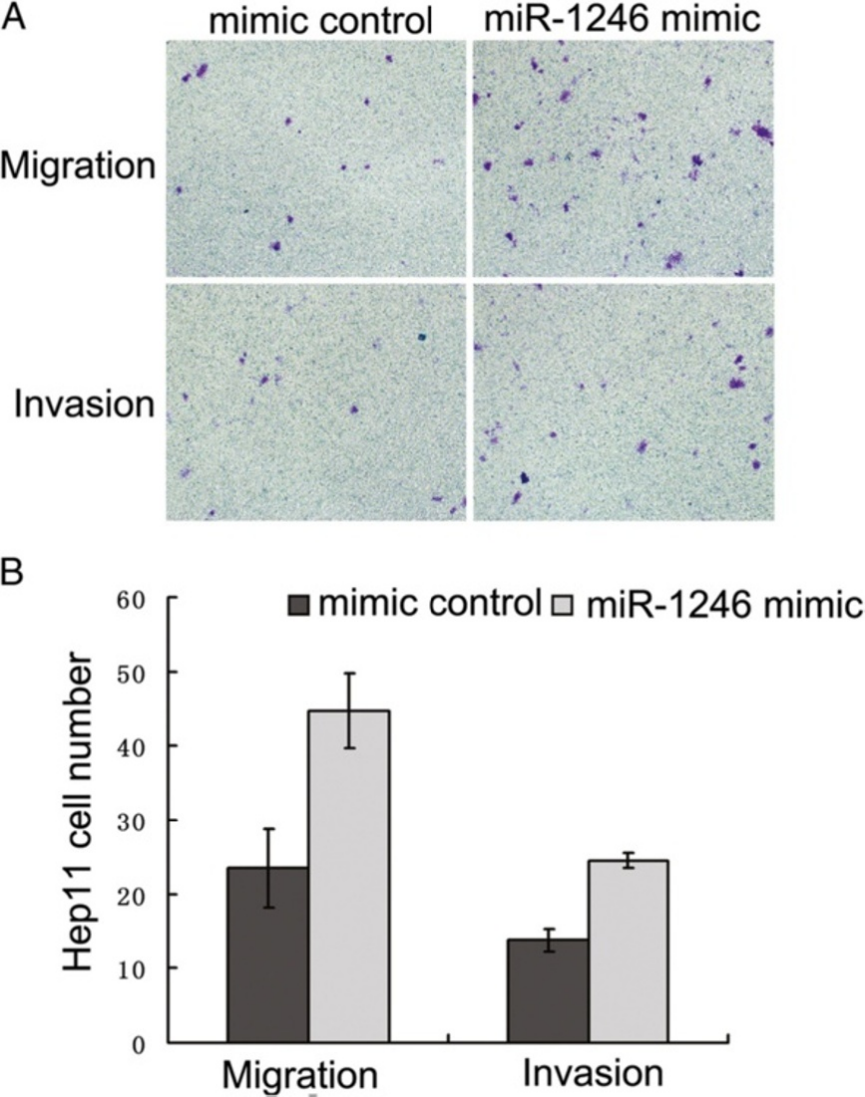
**Over-expression of miR-1246 promoted migration and invasion of Hep11. (A, B)** Transwell migration (n = 4) and invasion (n = 4) assays showed that Hep11 transfected with the miR-1246 mimic (800 nM) had more invasive and migratory potentials than the control (mimic control), **(A)** is a microscopic image of crystal violet staining; **(B)** shows the statistical results. Data represent the mean ± SD of four independent experiments. *P < 0.01.

The higher expression of miR-1246 in more metastatic HCC line Hep12 promoted us to investigate its effect on the migration and invasion of HCC. BEL7402 and SMMC7721 cells were transfected with either miR-1246 mimic, mimic control, miR-1246 inhibitor or inhibitor control and then subjected to cell migration assay and cell invasion assay, respectively As expected, cell motility in both cell lines was significantly reduced after transfection of the miR-1246 inhibitor compared with inhibitor control (Figure 1). Similar results were obtained in Hep12 cells. Their motility was significantly reduced after transfection of the miR-1246 inhibitor compared with inhibitor control (Additional file 4: Figure S3). However, no difference was observed between miR-1246 mimic and mimic control in HCC line SMMC7721 and BEL7402 (Additional file 5: Figure S4). We speculate that this unexpected phenomenon may be caused by the relatively high expression of miR-1246 in SMMC7721 and BEL7402. So Hep11 with low expression of miR-1246 was chosen to evaluate the effects of miR-1246 mimic. Compared with control group, Hep11 transfected with miR-1246 mimic have significantly higher migration and invasion capacity (Figure 2). These results suggested that down-regulation of miR-1246 impaired migration and invasion of HCC while up-regulation of miR-1246 promoted migration and invasion.

To further investigate the functional significance of miR-1246 in HCC, we performed MTS assays in SMMC7721 cells 1, 2 and 3 days after transfection with miR-1246 mimic, mimic control, miR-1246 inhibitor or inhibitor control. As demonstrated in Additional file 6: Figure S5, neither overexpression nor downregulation of miR-1246 obviously impaired the cell growth.

### miR-1246 directly inhibited CADM1 expression via its 3′UTR

**Figure 3:**
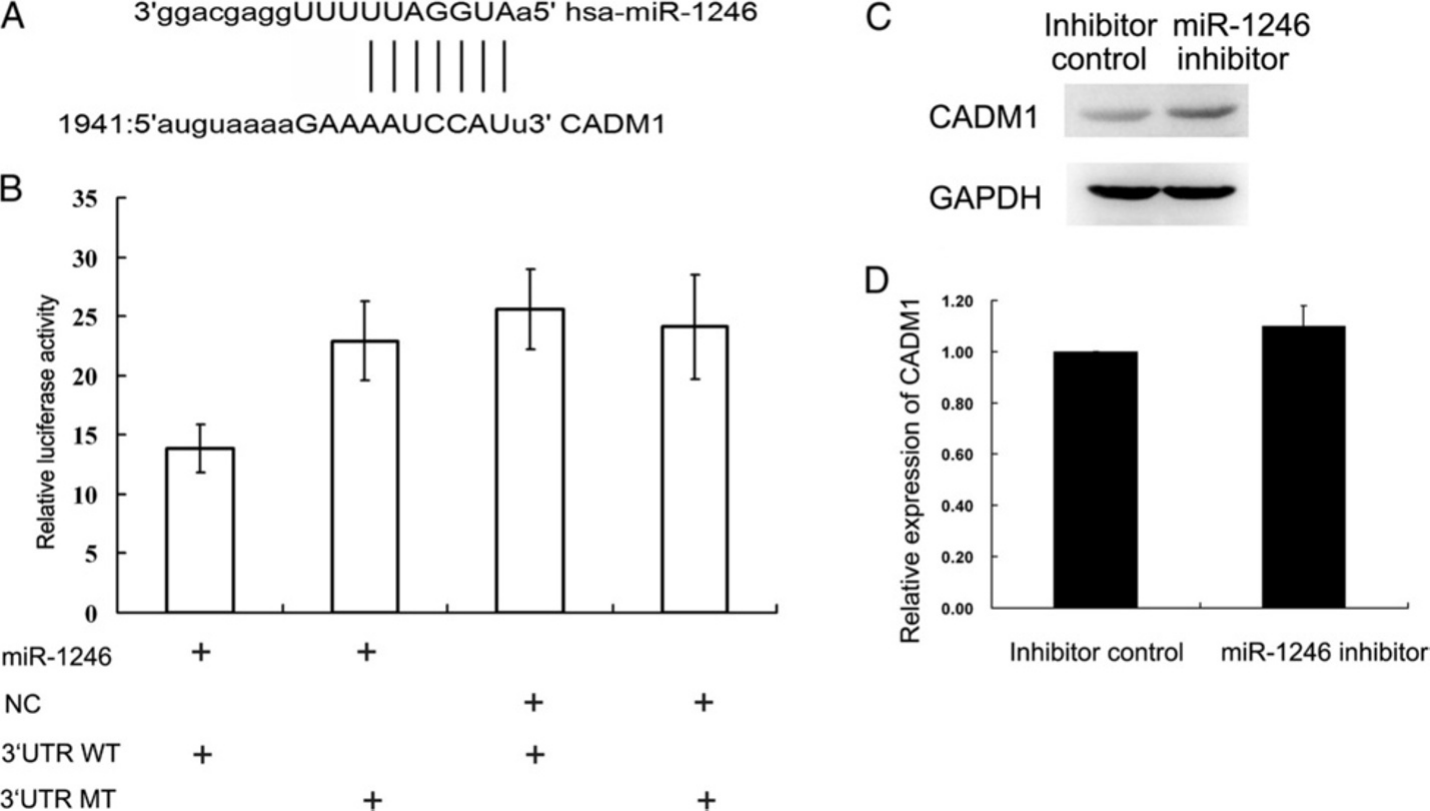
**CADM1 was a direct target of miR-1246. (A)** Putative binding site for miR-1246 in the 3′UTR of CADM1 was revealed by TargetScan. **(B)** The miR-1246 binding site on CADM1 3′UTR was confirmed by luciferase assay in 293 T cells after cotransfection with (i) a plasmid containing a fragment of CADM1 3′UTR that included either the wild type or mutant predicted miR-1246 binding site and (ii) the miR-1246 mimic or the mimic control. Data represent the mean ± SD of at least three independent experiments. *P < 0.01. **(C)** Western blot assay showed increased CADM1 expression in SMMC7721 cells after transfection with the miR-1246 inhibitor (800 nM). **(D)** Real time PCR showed that miR-1246 did not affect CADM1 mRNA expression.

To understand the mechanisms by which inhibition of miR-1246 reduced migration and invasion, we used bioinformatics analysis to identify miR-1246 targets. There was a conserved binding site of miR-1246 in the CADM1 3′UTR (Figure 3A). To test whether CADM1 is a target of miR-1246, we conducted a standard luciferase reporter assay in 293 T cells. 293 T cells were transfected with the luciferase construct CADM1-WT or CADM1-MT, along with the internal control vector pGL3 and either the miR-1246 mimic or the mimic control. The cells were harvested at 48 hours and analyzed for dual luciferase activity. The results showed that the renilla luciferase activity in CADM1-WT-transfected cells decreased by more than 40% in miR-1246 mimic-cotransfected cells compared with that in mimic control-cotransfected cells. In addition, site-directed mutation of the seed region offset the inhibitory effect of miR-1246 mimic (Figure 3B).

To determine whether miR-1246 could regulate the expression of CADM1 in HCC, we measured the RNA and protein levels of CADM1 in SMMC7721 cells that were transfected with the miR-1246 inhibitor or the inhibitor control. The results showed that expression of CADM1 remained unchanged at the mRNA level (Figure 3D). The CADM1 protein level was increased after transfection with the miR-1246 inhibitor (Figure 3C). In Hep11 cells, overexpression of miR-1246 resulted in downregulation of CADM1 protein levels (Additional file 7: Figure S6). CADM1 expression was not changed in SMMC7721 cells after transfection with the miR-1246 mimic (Additional file 8: Figure S7), which may explain why overexpression of miR-1246 in SMMC7721 had no effects on its migration and invasion. The regulatory effects of miR-1246 on CADM1 may have reached the maximal extent due to the relatively high expression of miR-1246 even before the transfection of miR-1246 mimic.

### Downregulation of CADM1 enhanced migration and invasion of HCC cell lines

**Figure 4:**
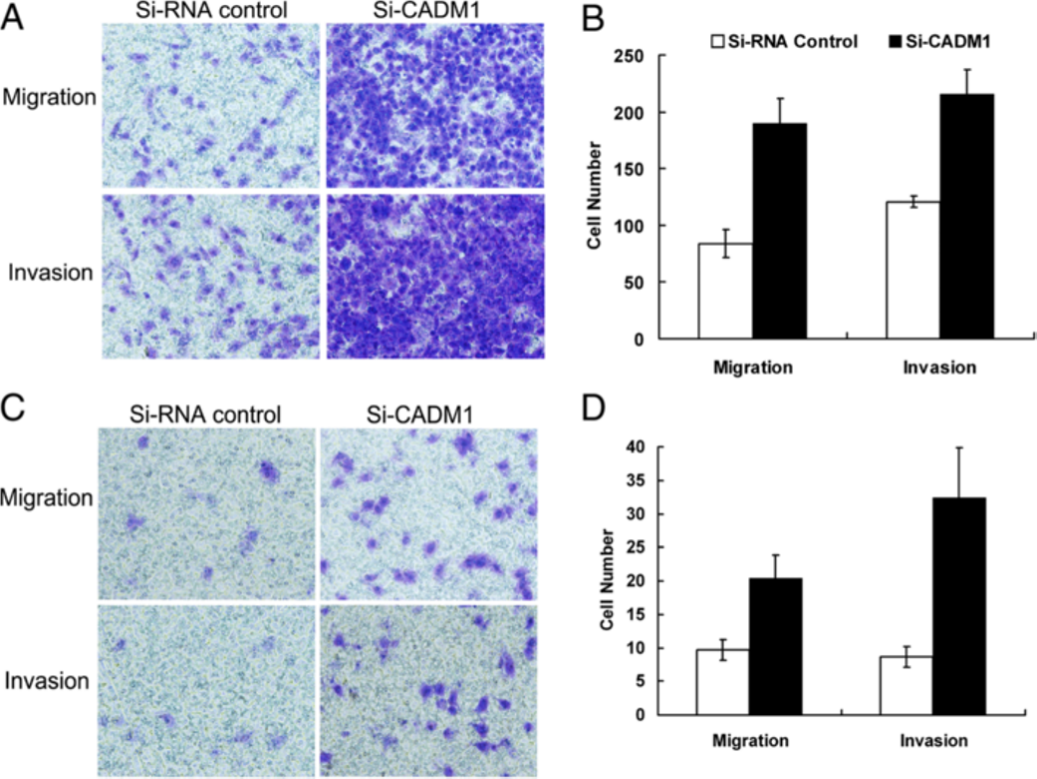
**CADM1 regulated HCC cell migration and invasion. (A, B)** Transwell migration (n = 4) and invasion (n = 4) assays showed that SMMC7721 cells that were transfected with the CADM1 siRNA (200 nM) had greater invasive and migratory potentials than the control (siRNA control). **(A)** is a microscopic image of Crystal violet staining; **(B)** shows the statistical results. **(C, D)** Transwell migration (n = 4) and invasion (n = 4) assays showed that BEL7402 cells that were transfected with the CADM1 siRNA (200 nM) had greater invasive and migratory potentials than the control (siRNA control). **(C)** is a microscopic image of Crystal violet staining; **(D)** shows the statistical results. Data represent the mean ± SD of four independent experiments. *P < 0.01.

We next evaluated whether downregulation of CADM1 levels might have a reverse effect on cell motility as observed following miR-1246 inhibition. Small interfering RNA (siRNA) designed to target at CADM1 was employed. Transfection of CADM1 siRNA caused a more than 50% reduction in CADM1 protein level compared to the Si-control (Additional file 9: Figure S8). We then compared expression of CADM1 in different HCC cell lines and found SMMC7721 and BEL7402 cells have high CADM1 expression levels (Additional file 10: Figure S9). These two cell lines were transfected with CADM1 siRNA and subjected to cell migration assay and cell invasion assay. As shown in Figure 4, cell motility was significantly enhanced after CADM1 downregulation compared with the control. Similar results were obtained in Hep11 cells (Additional file 11: Figure S10).

### Correlation between miR-1246 and CADM1 expression in liver cancer patients

**Figure 5:**
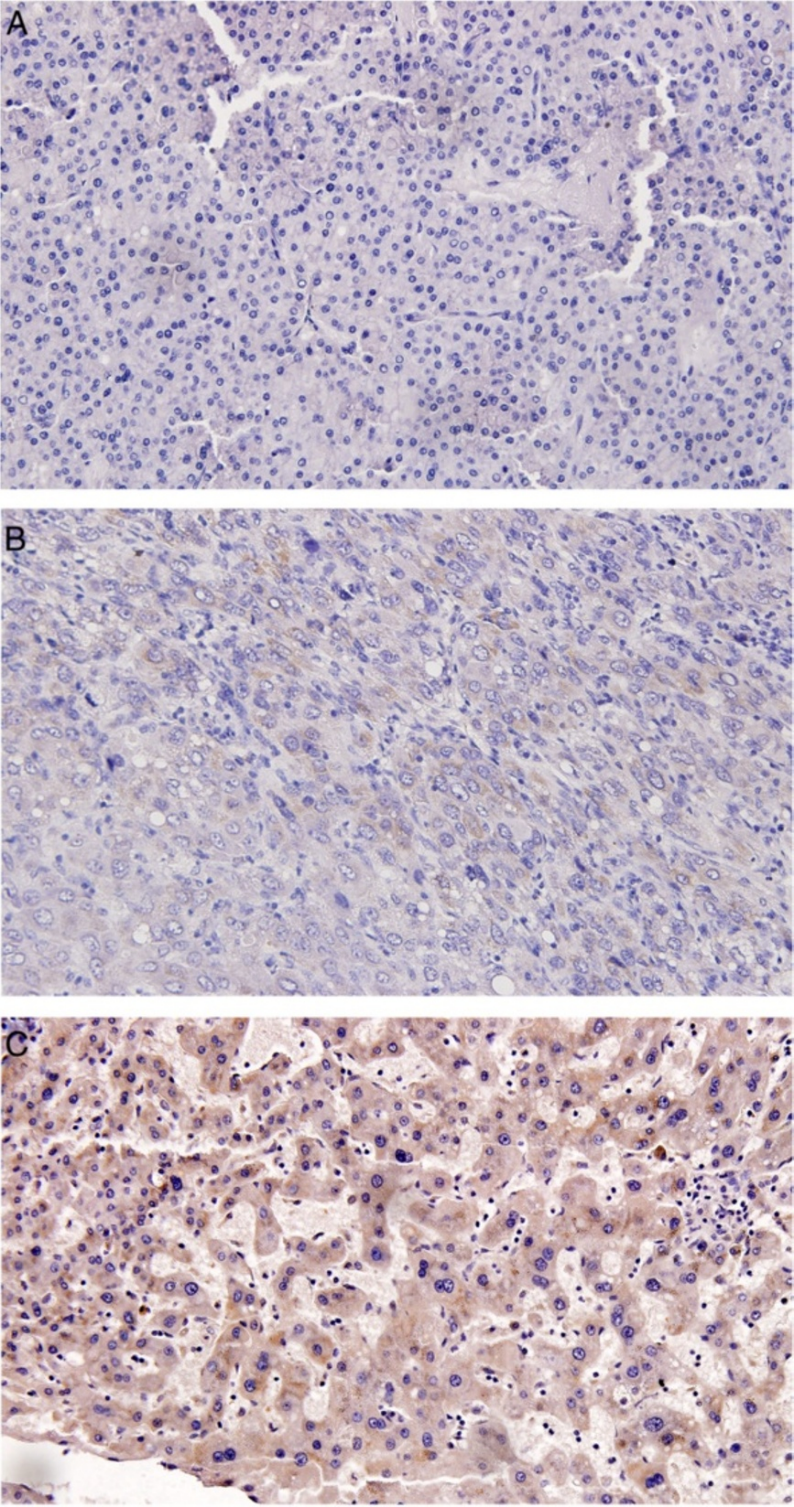
**Immunohistochemistry of CADM1. (A)** negative; **(B)** positive +; **(C)** positive++.

We obtained clinical samples from 38 patients of liver cancer. Additional file 1: TableS5 showed the clinical characteristics of these patients. To investigate the correlation between miR-1246 and CADM1 expression, we analyzed these liver cancer tissue samples by RT-PCR and immunohistochemisty for miR-1246 and CADM1, respectively. Figure 5 showed the characteristic expression of CADM1. The results demonstrated that patients with high miR-1246 expression were CADM1 negative while patients with low miR-1246 expression were CADM1 positive (p = 0.003 Additional file 1: Table S6). The expression of miR-1246 was negatively correlated to CADM1, which was of statistical significance.

### Correlation between miR-1246 and CADM1 expression and disease free survivol (DFS) of the patients

**Figure 6:**
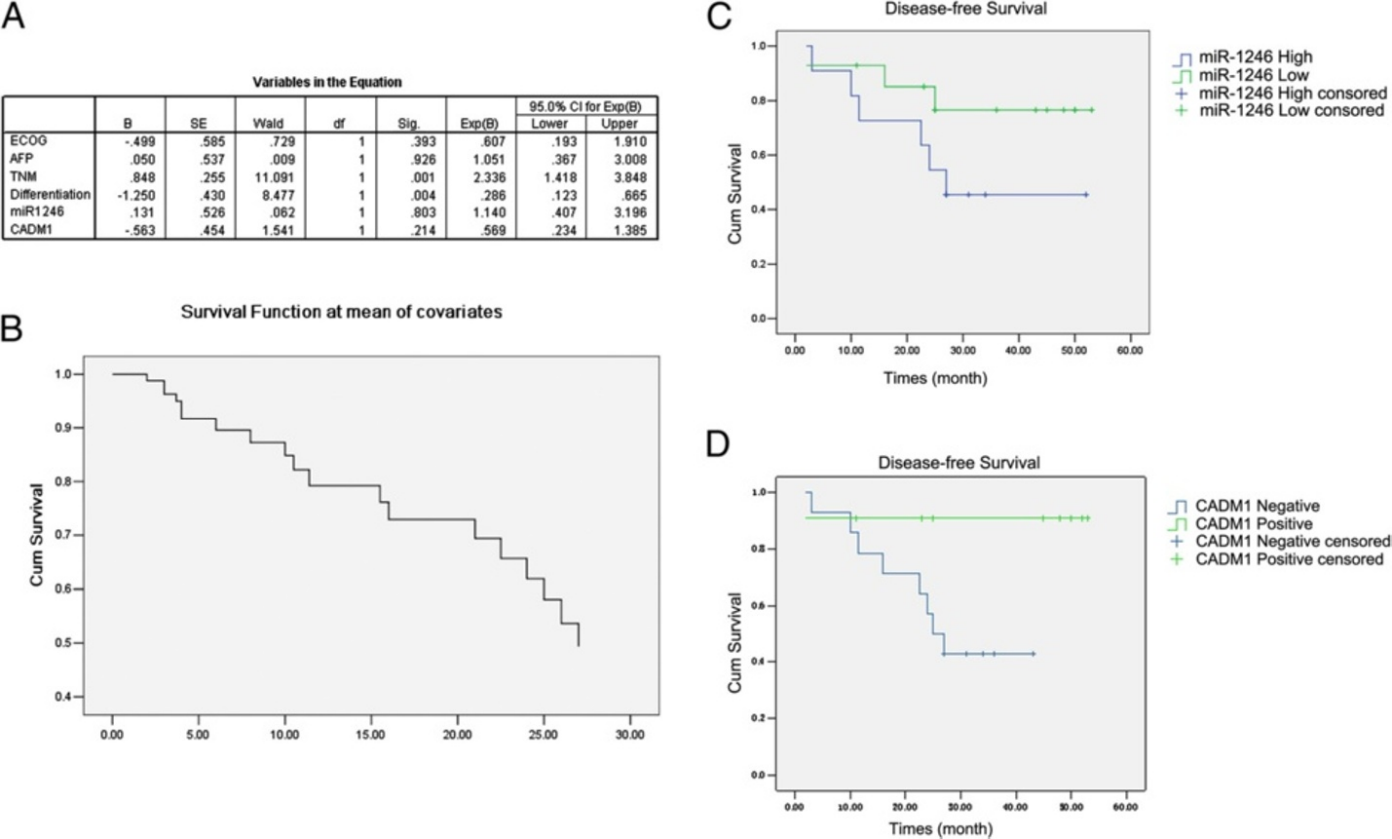
**Influence of different factors on DFS of the patients. (A)** Cox assay was used to examine the influence of different factors on DFS of the patients; **(B)** the DFS of all patients; **(C)** the influence of miR-1246 expression on DFS of stage I patients (p = 0.143); **(D)** the relationship between expression of and DFS in stage I patients (p = 0.039).

From 2009 to 2010, 91 patients with liver cancer got surgery in Peking Union Medical College hospital and among them only 38 had follow-up data for statistical analysis. By June 30^th^, 2013, 19 patients had recurrence and 11 patients died. So we didn’t perform OS. Using cox assay to analyze the influence of multiple factors on DFS such as ECOG score, serum AFP, TNM, pathological differentiation and miR-1246 and CADM1 expression, we found only TNM and pathological differentiation were statistically significantly correlated with DFS (Figure 6A, B), Kaplan-Meier assay also confirmed this result (Additional file 1: Table S7 and 8).

To rule out the influence of TNM on DFS, we analyzed 25 patients who were classified as stage 1 according to TNM. Patients with high miR-1246 expression had DFS of 32.53 m ± 5.69 m (21.37 m-41.30 m) while patients with low miR-1246 expression had DFS of 44.11 m ± 4.61 m (35.07 m-53.15 m) (p = 0.143 Figure 6C). Patients who were CADM1 positive had DFS of 48.36 m ± 4.42 m (39.69 m-57.02 m) while patients were CADM1 negative had DFS of 28.35 m ± 3.76 m (20.97-35.72) (p = 0.039 Figure 6D). Since miR-1246 and CADM1 were correlated, we draw the conclusion that higher miR-1246 expression combined with low CADM1 expression could serve as a risk factor for stage1 liver cancer patients. We didn’t analyze patients in stage 2 and 3 due to the limited patient number.

## Discussion

Tumor metastasis is a complex series of steps that involve a number of influential factors. Additionally, due to tumor heterogeneity, the mechanisms underlying distal metastasis could be totally different even though primary tumors possess similar clinical manifestations and histological types. Thus, the advance of detecting biomarker indicative of high-risk tumor metastasis may immensely benefit the approach of personalized cancer treatment. Lately, miRNA has been regarded as an excellent biomarker owing to several unique features: an average 22 nucleotides in length, more stable expression compared with mRNA and more likely to be detected in samples with mRNA and protein degraded. Furthermore, the convenience of synthesizing an overexpressed or an interfering sequence also made miRNA a potential candidate for novel therapeutic strategies.

The expression level of miR-1246 in HCC cells is quite high [13]. Our previous study analyzed the differential expression of miRNAs between Hep11 and Hep12 using microRNA microarray [13] and discovered differential expression of miR-1246. To define its role, we carried out several biological function assays in different HCC cell lines and found that inhibition of miR-1246 in SMMC7721 and BEL7402 which have relatively high expression levels of miR-1246 significantly reduce migration and invasion while overexpression of miR-1246 had little effects. We speculate that this may be attributed to the abundant expression levels of miR-1246 in these HCC cell lines and that regulation of target genes by miR-1246 may have reached the maximal extent. For Hep11 which has low levels of miR-1246, transfection with miR-1246 mimic could significantly promote migration and invasion. Variation of miR-1246 levels had no effect on HCC cell proliferation.

According to the prediction of biological databases, CADM1 might be a target gene of miR-1246. *CADM1*, also known as *TSLC1*, is a well-defined tumor suppressor gene that has been discovered recently. *CADM1* gene encodes a 442-amino acid protein which contains an extracellular domain, a transmembrane domain and a cytoplasmic domain. Extracellular domain of CADM1 of 373 amino acids includes three Ig-like C2-type domain connected by disulfide bonds. This structure is also existed in other immunoglobulin superfamily cell adhesion molecule (IgCAM) members, which are refered to as nectins [15–17]. Therefore, CADM1 is considered to be involved in cell-cell interactions. Expression of CADM1 is lost or reduced in a variety of cancers, including non-small cell lung cancer (NSCLC) [18, 19], breast cancer [20], cervix cancer [21, 22], and HCC [23, 24]. This reduction has been associated with enhanced metastasis potential and poor prognosis. So far, it has been postulated that loss of heterozygosity (LOH) [17], promoter hypermethylation [18, 19, 24] and miRNA mediated regulation might be mechanisms underlying the loss of CADM1 expression. miR-10b and miR-216a are two microRNAs implicated in regulation of CADM1 in HCC [23, 25]. Li et al. reported that miR-10b enhances tumor invasion and metastasis through targeting CADM1. Moreover, patients with higher miR-10b expression had significantly poorer overall survival.

Although higher expression of miR-1246 has been reported in the serum of tumor carrying mice [26] and oesophageal squamous cell carcinoma [27], few studies are available for interpreting miR-1246’s function in tumor. Our study is the first to report miR-1246 could regulate invasion and migration of HCC cell via targeting CADM1. There is no doubt that CADM1 functions as a tumor suppressor gene in HCC [23, 28]. In this study, we also demonstrated that CADM1 RNA interference results in up-regulation of invasion and migration in HCC cell lines. However, the mechanism underlying tumor suppression by CADM1 remains to be fully elucidated.

We confirmed that miR-1246 could promote migration and invasion through CADM1 in HCC cell lines. Whether miR-1246 and CADM1 expression are correlated in tumor tissues is not investigated before. Here, using clinical samples from 38 patients of liver cancer, we analyzed miR-1246 and CADM1 expression by RT-PCR and immunohistochemisty, respectively and found that miR-1246 expression was negatively correlated to CADM1, which was of statistical significance. We also analyzed the influence of multiple factors on DFS such as ECOG score, serum AFP, TNM, pathological differentiation and miR-1246 and CADM1 expression and found only TNM and pathological differentiation were statistically significantly correlated with DFS. In 25 patients who were classified as stage 1 according to TNM, those who were CADM1 positive had long DFS while patients were CADM1 negative had short DFS and the difference was statistically significant. Patients who had high miR-1246 expression had short DFS while those with low miR-1246 expression had long DFS, although the difference was not statistically significant. When we analyze miR-1246 expression, we use the total RNA extracted from the tumor tissues which contain not only epithelial cancer tissues, but also meschymal cancer tissues. Since the proportion of epithelial cancer tissues in tumors differs between patients, analyzing miR-1246 expression in total RNA might influence the results. CADM1 expression is detected by immunohistochemistry which is more accurate because pathologists can directly determine the expression of CADM1 in tumor tissues. In our study, miR-1246 expression is negatively correlated with CADM1. So although the correlation between miR-1246 and DFS is not statistically significant, high miR-1246 expression combined with low CADM1 could still serve as a risk factor for DFS.

## Conclusions

In this study, we showed that miR-1246 is highly expressed in more metastatic HCC cells and inhibition of miR-1246 effectively reduced migration and invasion of HCC cells by down-regulation CADM1. More importantly, we found high expression of miR-1246 combined with low expression of CADM1 might serve as a risk factor for stage1 liver cancer patients. Here, we provide new insights into the metastasis enhancer functions of miR-1246 in hepatocellular carcinoma. Identifying small molecules targeting miR-1246 might lead to vigorous therapeutic strategies for hepatocellular carcinoma.

## Electronic supplementary material

Additional file 1: Table S1: The sequences of siRNAs of CADM1. **Table S2**: The primer (mRNA) of real time PCR. **Table S3**: The primer (miRNA) of real time PCR. **Table S4**: The sequences of target gene (CADM1 3′UTR) and mutation target gene. **Table S5**: The basic condition of patients. **Table S6**: The relationship between miR1246 and CADM1 p=0.003. **Table S7**: The relationship between TNM and DFS p=0.011. **Table S8**: The relationship between differentiation and DFS p=0.016. (DOC 61 KB)

Additional file 2: Figure S1: Relative expression miR-1246 in Hep11 and Hep12. (TIFF 93 KB)

Additional file 3: Figure S2: Relative expression miR-1246 in different HCC cell lines. (TIFF 128 KB)

Additional file 4: Figure S3: Inhibition of miR-1246 reduced migration and invasion of Hep12. Transwell migration (n=4) and invasion (n=4) assays showed that Hep12 cells transfected with the miR-1246 inhibitor (800 nM) had lower invasive and migratory potentials than the control (inhibitor control). **(A)** is a microscopic image of crystal violet staining; **(B)** shows the statistical results. (TIFF 4 MB)

Additional file 5: Figure S4: Upregulation of miR-1246 had no effect on migration and invasion of SMMC7721 and BEL7402. **(A, B)** Transwell migration (n=4) and invasion (n=4) assays showed that SMMC7721 cells transfected with the miR-1246 mimc (800 nM) and transfected with mimic control had no significant difference in invasive and migratory potentials **(A)** is a microscopic image of crystal violet staining; **(B)** shows the statistical results. **(C, D)** Transwell migration (n=4) and invasion (n=4) assays showed that BEL7402 cells transfected with the miR-1246 mimc (800 nM) and transfected with mimic control had no significant difference in invasive and migratory potentials **(C)** is a microscopic image of crystal violet staining; **(D)** shows the statistical results. Data represent the mean ± SD of four independent experiments. (TIFF 8 MB)

Additional file 6: Figure S5: miR-1246 had no effect on HCC cell proliferation. SMMC7721 cells were transfected with miR-1246 mimic, mimic control, miR-1246 inhibitor or inhibitor control. Cell numbers were determined with the MTS assay after 0, 1, 2 and 3 days. Data represent the mean ± SD of 6 independent experiments. (TIFF 2 MB)

Additional file 7: Figure S6: Western blot assay of CADM1 expression after overexpression of miR-1246 in Hep11 cells. (TIFF 509 KB)

Additional file 8: Figure S7: CADM1 protein level was not changed in SMMC7721 cells transfected with the miR-1246 mimic (800 nM) as compared to mimic control. (TIFF 864 KB)

Additional file 9: Figure S8: Western blot assay showed that CADM1 were downregulated in SMMC7721 cells transfected with the CADM1 siRNA (800 nM). The siRNA pool includes CADM1 siRNA 597, 659 and 1016. (TIFF 2 MB)

Additional file 10: Figure S9: Western blot assay of CADM1 expression in different HCC cell lines. (TIFF 913 KB)

Additional file 11: Figure S10: CADM1 knockdown in Hep11 promote migration and invasion. **(A)** Western blot assay showed that CADM1 were downregulated in Hep11 cells transfected with the CADM1 siRNA. **(B, C)** Transwell migration (n=4) and invasion (n=4) assays showed that Hep11 cells transfected with the CADM1 siRNA had greater invasive and migratory potentials than the control (siRNA control). **(B)** is a microscopic image of crystal violet staining; **(C)** shows the statistical results. (TIFF 4 MB)

## Abbreviations

HCC: Hepatocellular carcinoma
HBV: Hepatitis B virus
FBS: Fetal calf serum
qRT-PCR: Quantitative reverse transcription polymerase chain reaction
WT: Wild type
MT: Mutant type
CADM1: Cell adhesion molecule 1.
