# Supplementary material for: MicroRNA-1246 enhances migration and invasion through CADM1 in hepatocellular carcinoma
Source: BMC Cancer. 2014 Aug 27;14:616. doi: 10.1186/1471-2407-14-616 (PMC4150976; doi:10.1186/1471-2407-14-616)
Supplement: Supplementary file 1 — Additional file 1: Table S1: The sequences of siRNAs of CADM1. Table S2: The primer (mRNA) of real time PCR. Table S3: The primer (miRNA) of real time PCR. Table S4: The sequences of target gene (CADM1 3′UTR) and mutation target gene. Table S5: The basic condition of patients. Table S6: The relationship between miR1246 and CADM1 p=0.003. Table S7: The relationship between TNM and DFS p=0.011. Table S8: The relationship between differentiation and DFS p=0.016. (DOC 61 KB) [file 12885_2014_4790_MOESM1_ESM.doc]

Additional file

Table S1 The sequences of siRNAs of CADM1.

| RNA Name | Sequence(enter all sequence 5' to 3') |
| --- | --- |
| CADM1 siRNA_597 | GGUGGAAGGUGAGGAGAUUTT |
| CADM1 siRNA_659 | UCAGGUGGUUCAAAGGGAATT |
| CADM1 siRNA_1016 | CCAACCUGUUCAUCAAUAATT |

Table S2 The primer (mRNA) of real time PCR

| Gene | Forward primer(5'→3') | Reverse primer(5'→3') |
| --- | --- | --- |
| CADM1 | TCAACACGCCGTACTGTCTG | GTGGGAGGAGGGATAGTTGTG |
| GAPDH | GGTCACCAGGGCTGCTTTTA | GAGGGATCTCGCTCCTGGA |

Table S3 The primer (miRNA) of real time PCR

| MiRNA | Primer Sequence(5'→3') |
| --- | --- |
| hsa-miR-1246 | AATGGATTTTTGGAGCAGG |
| U6 | CAAGGATGACACGCAAATTCG |

Table S4 The sequences of target gene (CADM1 3’UTR) and mutation target gene.

| Target gene  (CADM1) | F | GGGGGCCTTTTGGGAACGGACTGGTAATGTAAAAGAAAATCCATTATCGAGCAGCATTTTATTTAC |
| --- | --- | --- |
|  | R | GTAAATAAAATGCTGCTCGATAATGGATTTTCTTTTACATTACCAGTCCGTTCCCAAAAGGCCCCC |
| CADM1  mutation | F | GGGGGCCTTTTGGGAACGGACTGGTATAATAACAATGCAGAACACATCGAGCAGCATTTTATTTAC |
|  | R | GTAAATAAAATGCTGCTCGATGTGTTCTGCATTGTTATTATACCAGTCCGTTCCCAAAAGGCCCCC |

Table S5 The basic condition of patients

|  |  | miR-1246 High | miR-1246 Low | P1  Value | CADM1 Negative | CADM1 Positive + | CADM1 Positive ++ | p2 value |
| --- | --- | --- | --- | --- | --- | --- | --- | --- |
| Gender | M  F | 14  1 | 16  7 | 0.114 | 14  2 | 11  4 | 5  2 | 0.542 |
| Age | ≤65  >65 | 13  2 | 17  6 | 0.440 | 14  2 | 13  2 | 3  4 | **0.035** |
| TNM | 1  2  3  4 | 11  2  1  1 | 14  2  6  1 | 0.503 | 14  0  0  2 | 7  4  4  0 | 4  0  3  0 | **0.01** |
| ECOG | 0  1 | 4  11 | 14  9 | 0.052 | 5  11 | 7  8 | 6  1 | 0.055 |
| AFP | ≤200  >200 | 4  11 | 10  13 | 0.329 | 6  10 | 4  11 | 4  3 | 0.385 |
| Differentiation | L  M  H | 7  6  2 | 10  7  6 | 0.617 | 7  7  2 | 8  4  3 | 2  2  3 | 0.471 |

Table S6 The relationship between miR1246 and CADM1 p=0.003

|  | CADM1 negative | CADM1 positive+ | CADM1 positive++ | Summation |
| --- | --- | --- | --- | --- |
| miR-1246 High | 11 | 3 | 1 | 15 |
| miR-1246 Low | 5 | 12 | 6 | 23 |
| Summation | 16 | 15 | 7 |  |

Table S7 The relationship between TNM and DFS p=0.011

|  | Mean | | | |
| --- | --- | --- | --- | --- |
|  | Estimate | Std. Error | 95% Confidence Interval | |
| TNM |  |  | Lower Bound | Upper Bound |
| I | 38.08 | 3.86 | 31.25 | 46.37 |
| II | 17.13 | 4.28 | 8.73 | 25.52 |
| III | 20.24 | 6.82 | 6.88 | 33.06 |
| IV | 7.25 | 3.25 | 0.88 | 13.62 |
| Overall | 32.28 | 3.45 | 25.53 | 39.04 |

Table S8 The relationship between differentiation and DFS p=0.016

|  | Mean | | | |
| --- | --- | --- | --- | --- |
|  | Estimate | Std. Error | 95% Confidence Interval | |
| Differentiation |  |  | Lower Bound | Upper Bound |
| Low | 22.58 | 4.99 | 12.78 | 32.38 |
| Middle | 32.53 | 4.77 | 23.17 | 41.88 |
| High | 48.43 | 3.31 | 41.95 | 54.91 |
| Overall | 32.28 | 3.45 | 25.53 | 39.04 |
