# Supplementary figures and images for: MicroRNA-1246 enhances migration and invasion through CADM1 in hepatocellular carcinoma
Source: BMC Cancer. 2014 Aug 27;14:616. doi: 10.1186/1471-2407-14-616 (PMC4150976; doi:10.1186/1471-2407-14-616)

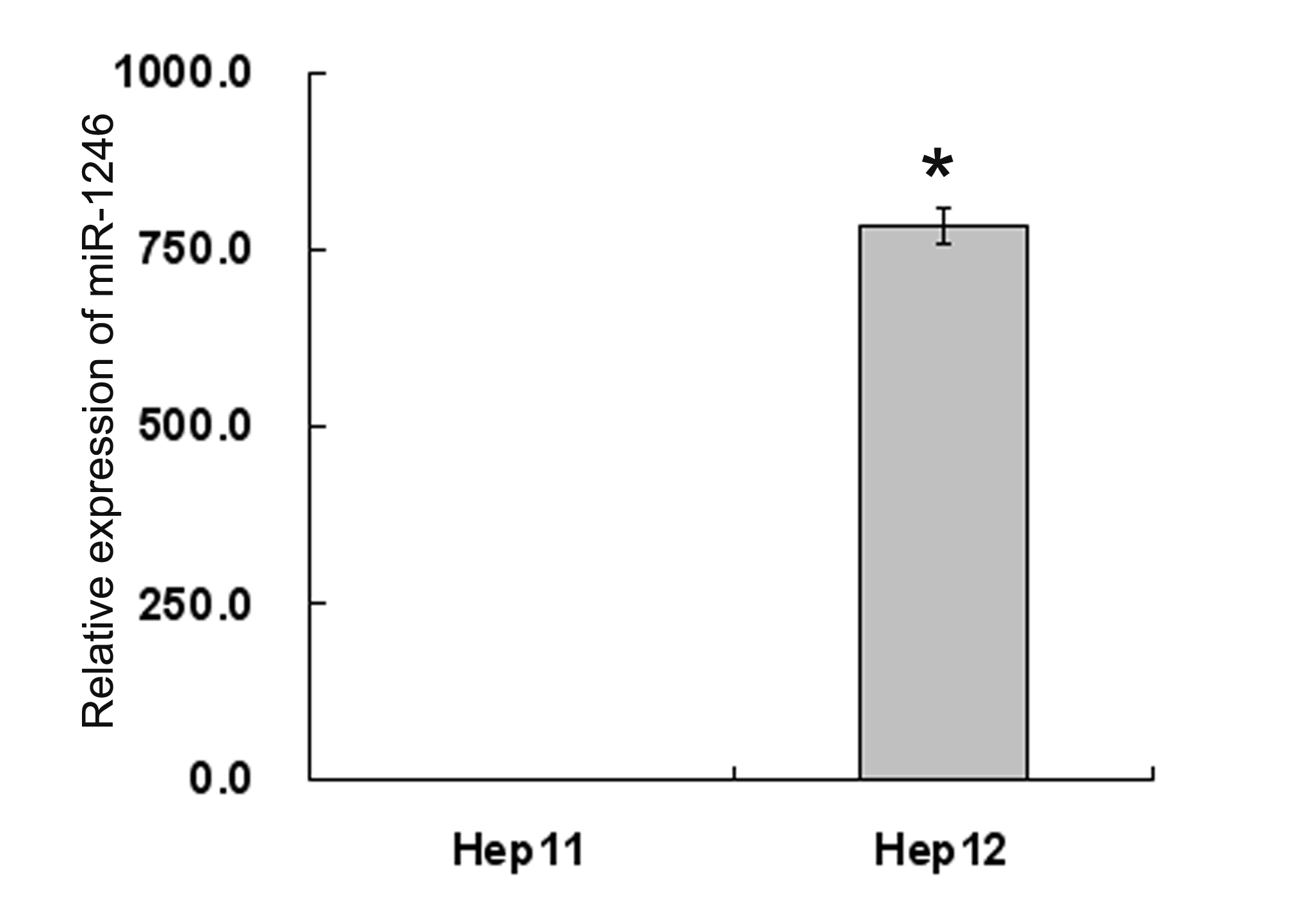

Supplement: Supplementary file 2 — Additional file 2: Figure S1: Relative expression miR-1246 in Hep11 and Hep12. (TIFF 93 KB) [file 12885_2014_4790_MOESM2_ESM.tiff]

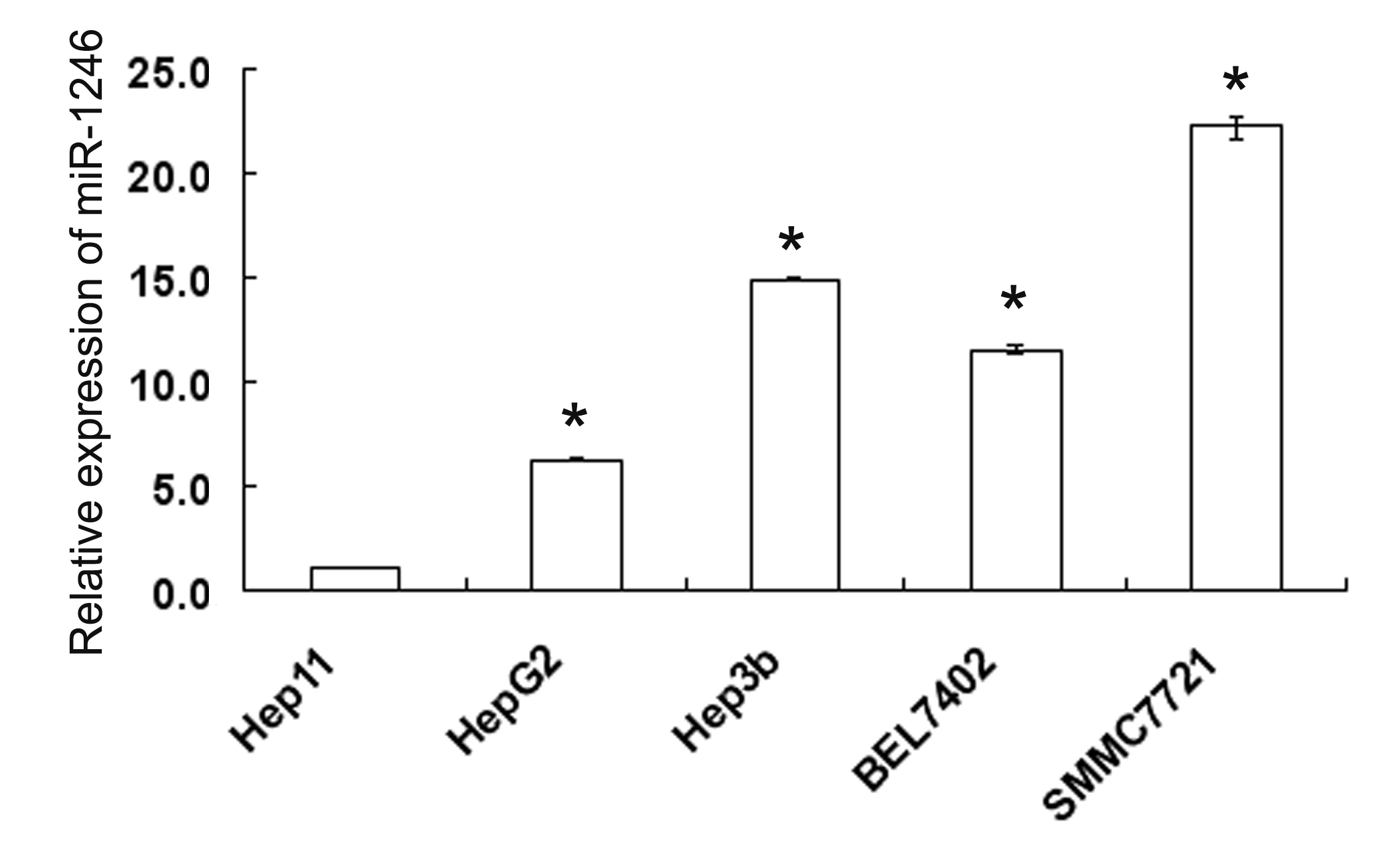

Supplement: Supplementary file 3 — Additional file 3: Figure S2: Relative expression miR-1246 in different HCC cell lines. (TIFF 128 KB) [file 12885_2014_4790_MOESM3_ESM.tiff]

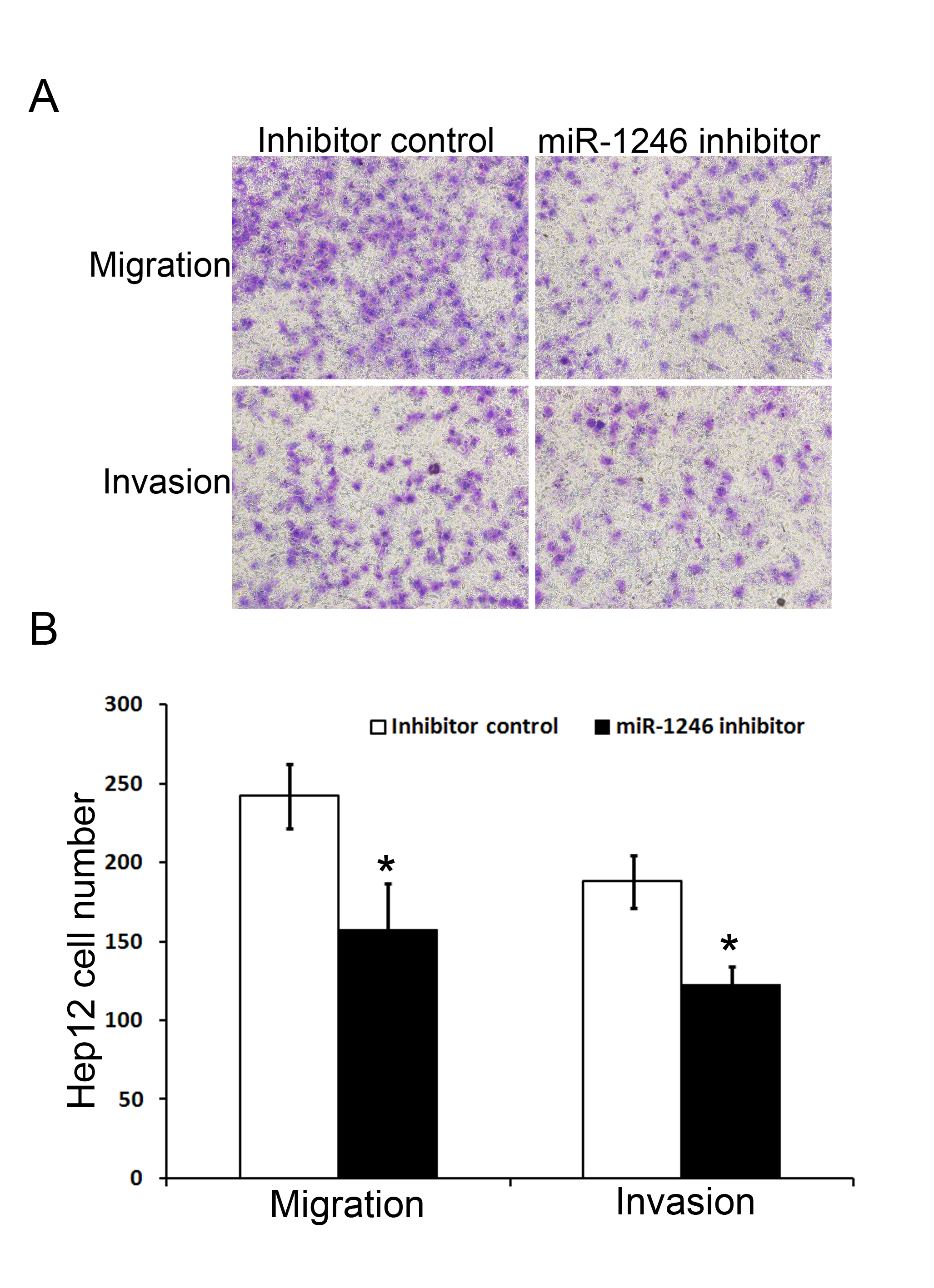

Supplement: Supplementary file 4 — Additional file 4: Figure S3: Inhibition of miR-1246 reduced migration and invasion of Hep12. Transwell migration (n=4) and invasion (n=4) assays showed that Hep12 cells transfected with the miR-1246 inhibitor (800 nM) had lower invasive and migratory potentials than the control (inhibitor control). (A) is a microscopic image of crystal violet staining; (B) shows the statistical results. (TIFF 4 MB) [file 12885_2014_4790_MOESM4_ESM.tiff]

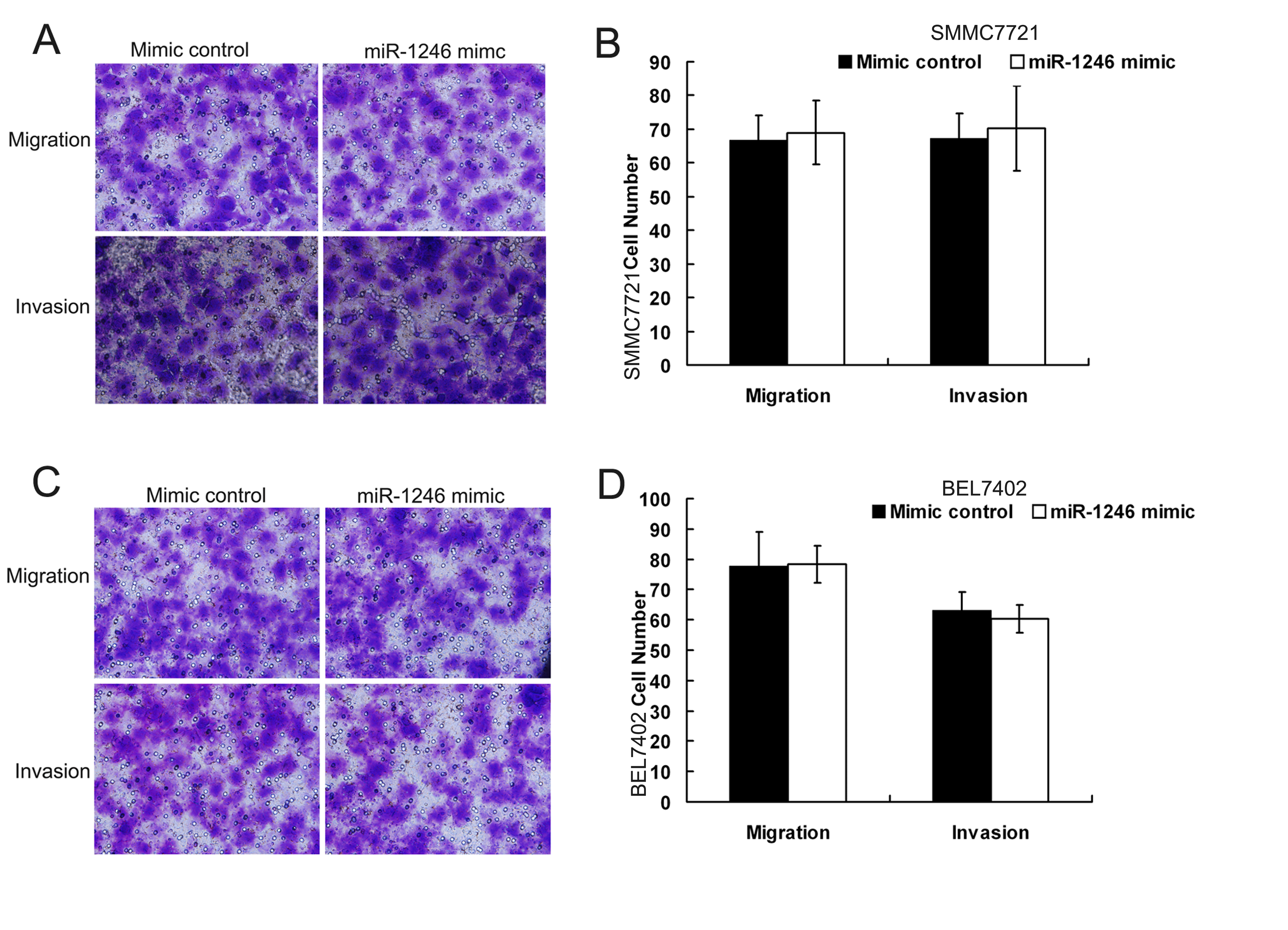

Supplement: Supplementary file 5 — Additional file 5: Figure S4: Upregulation of miR-1246 had no effect on migration and invasion of SMMC7721 and BEL7402. (A, B) Transwell migration (n=4) and invasion (n=4) assays showed that SMMC7721 cells transfected with the miR-1246 mimc (800 nM) and transfected with mimic control had no significant difference in invasive and migratory potentials (A) is a microscopic image of crystal violet staining; (B) shows the statistical results. (C, D) Transwell migration (n=4) and invasion (n=4) assays showed that BEL7402 cells transfected with the miR-1246 mimc (800 nM) and transfected with mimic control had no significant difference in invasive and migratory potentials (C) is a microscopic image of crystal violet staining; (D) shows the statistical results. Data represent the mean ± SD of four independent experiments. (TIFF 8 MB) [file 12885_2014_4790_MOESM5_ESM.tiff]

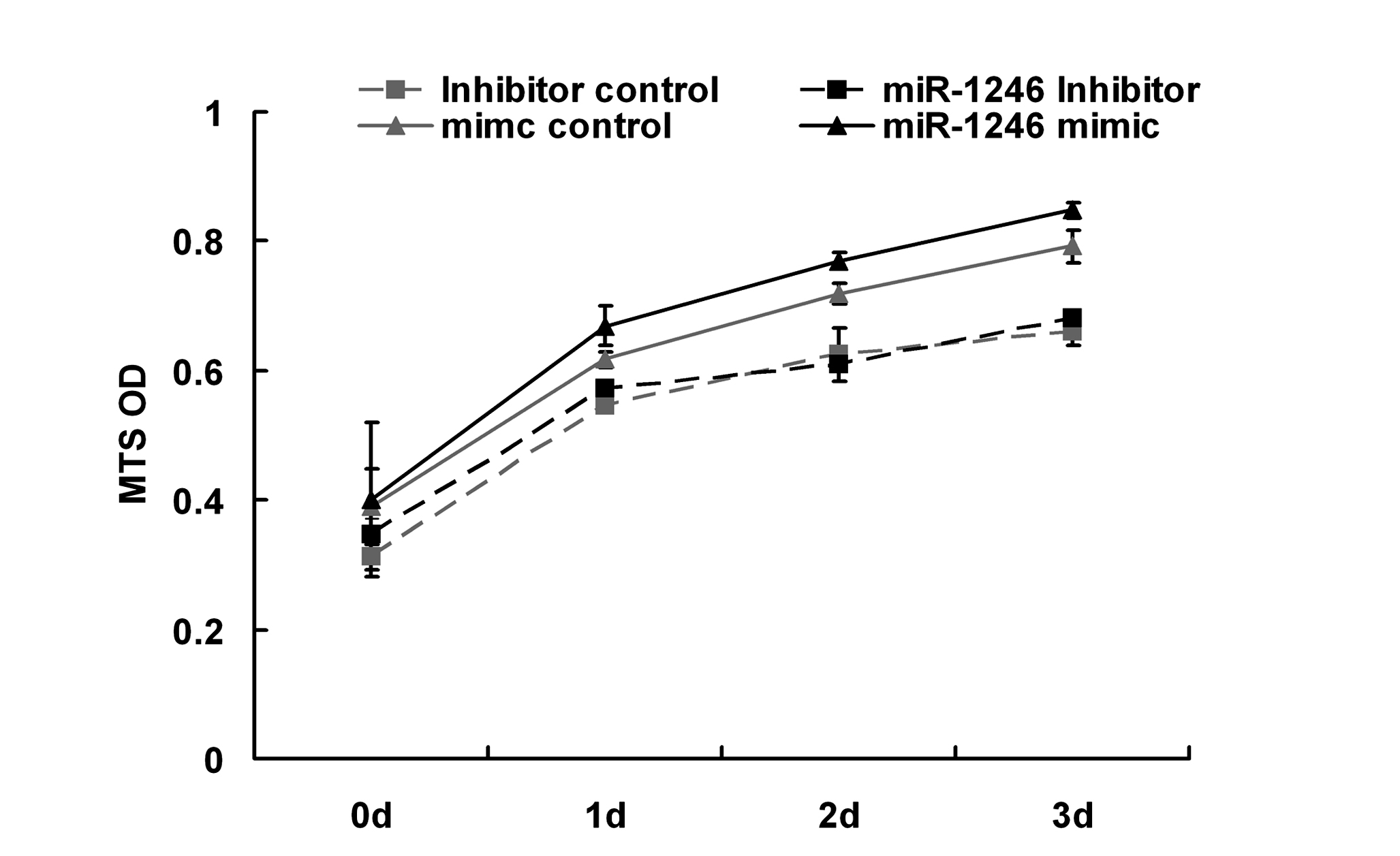

Supplement: Supplementary file 6 — Additional file 6: Figure S5: miR-1246 had no effect on HCC cell proliferation. SMMC7721 cells were transfected with miR-1246 mimic, mimic control, miR-1246 inhibitor or inhibitor control. Cell numbers were determined with the MTS assay after 0, 1, 2 and 3 days. Data represent the mean ± SD of 6 independent experiments. (TIFF 2 MB) [file 12885_2014_4790_MOESM6_ESM.tiff]

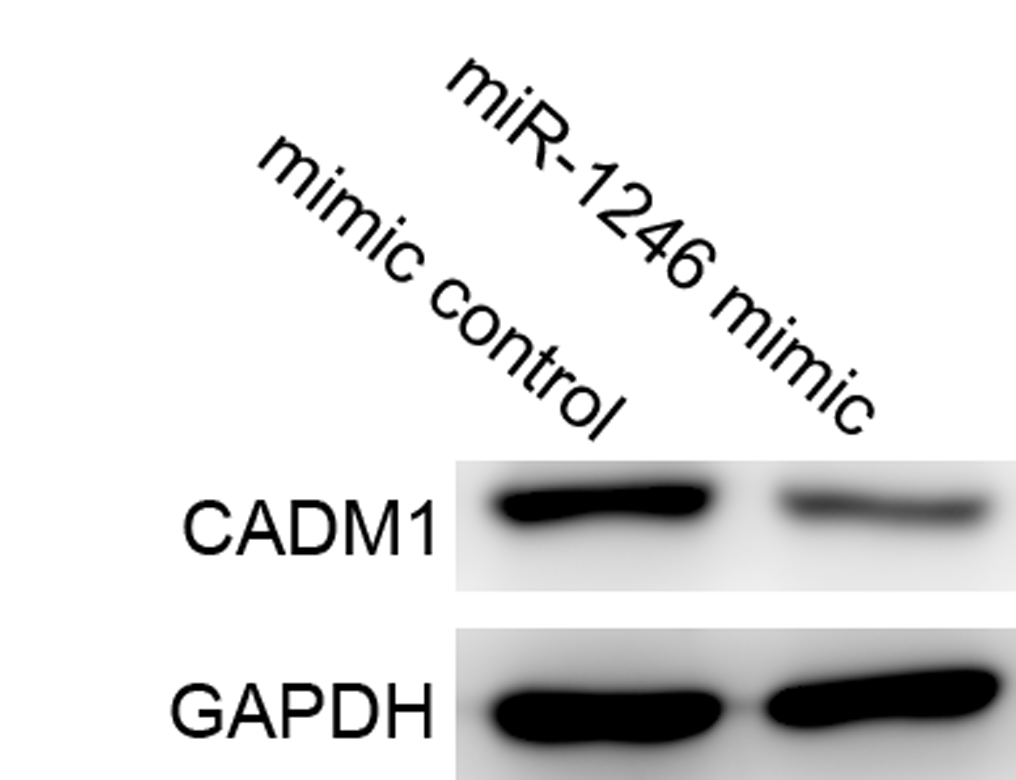

Supplement: Supplementary file 7 — Additional file 7: Figure S6: Western blot assay of CADM1 expression after overexpression of miR-1246 in Hep11 cells. (TIFF 509 KB) [file 12885_2014_4790_MOESM7_ESM.tiff]

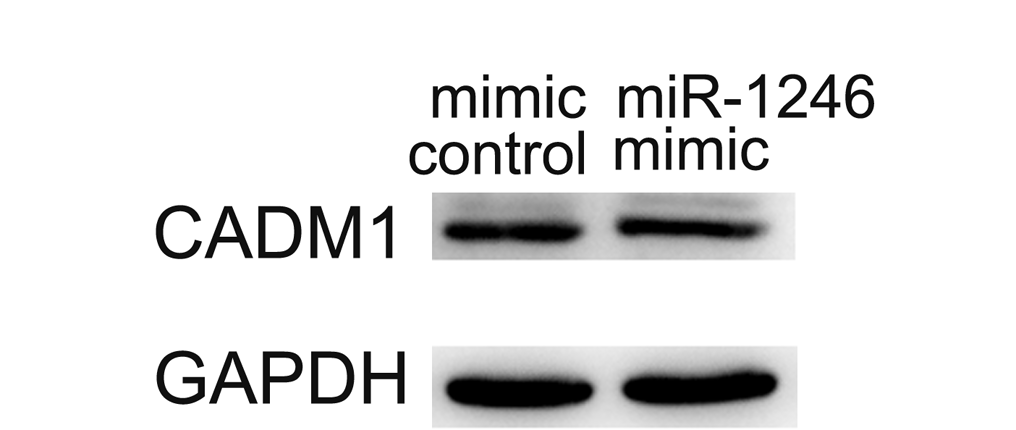

Supplement: Supplementary file 8 — Additional file 8: Figure S7: CADM1 protein level was not changed in SMMC7721 cells transfected with the miR-1246 mimic (800 nM) as compared to mimic control. (TIFF 864 KB) [file 12885_2014_4790_MOESM8_ESM.tiff]

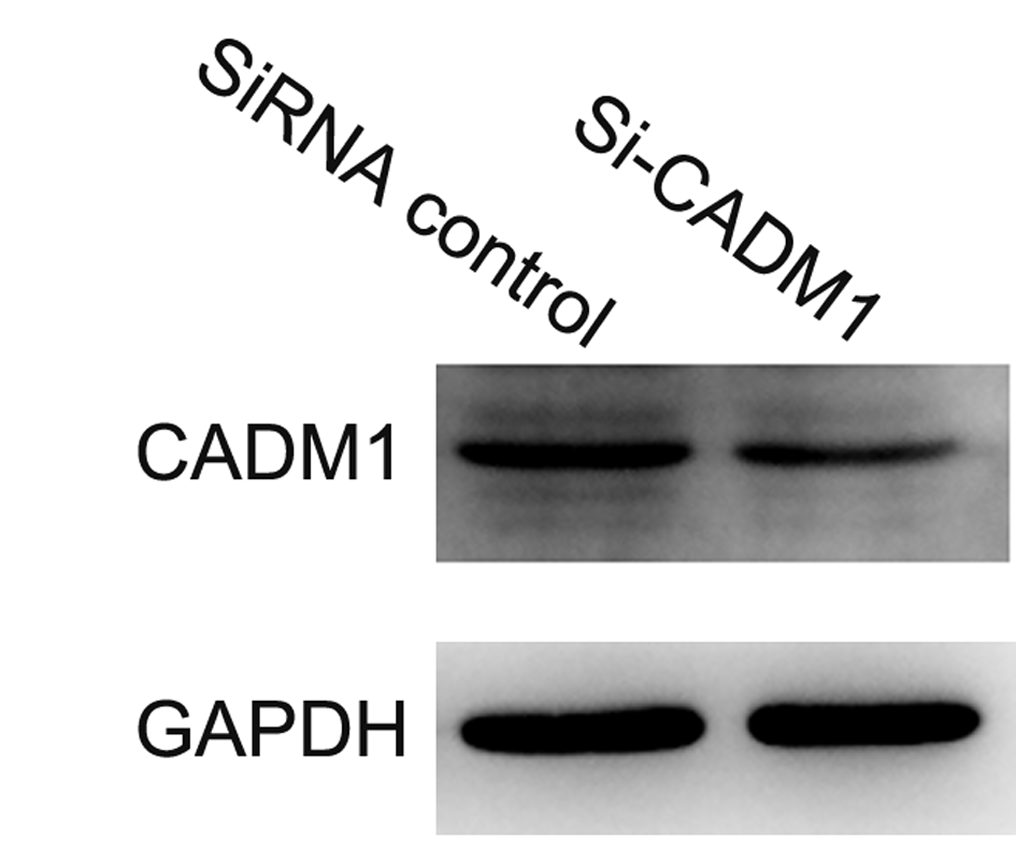

Supplement: Supplementary file 9 — Additional file 9: Figure S8: Western blot assay showed that CADM1 were downregulated in SMMC7721 cells transfected with the CADM1 siRNA (800 nM). The siRNA pool includes CADM1 siRNA 597, 659 and 1016. (TIFF 2 MB) [file 12885_2014_4790_MOESM9_ESM.tiff]

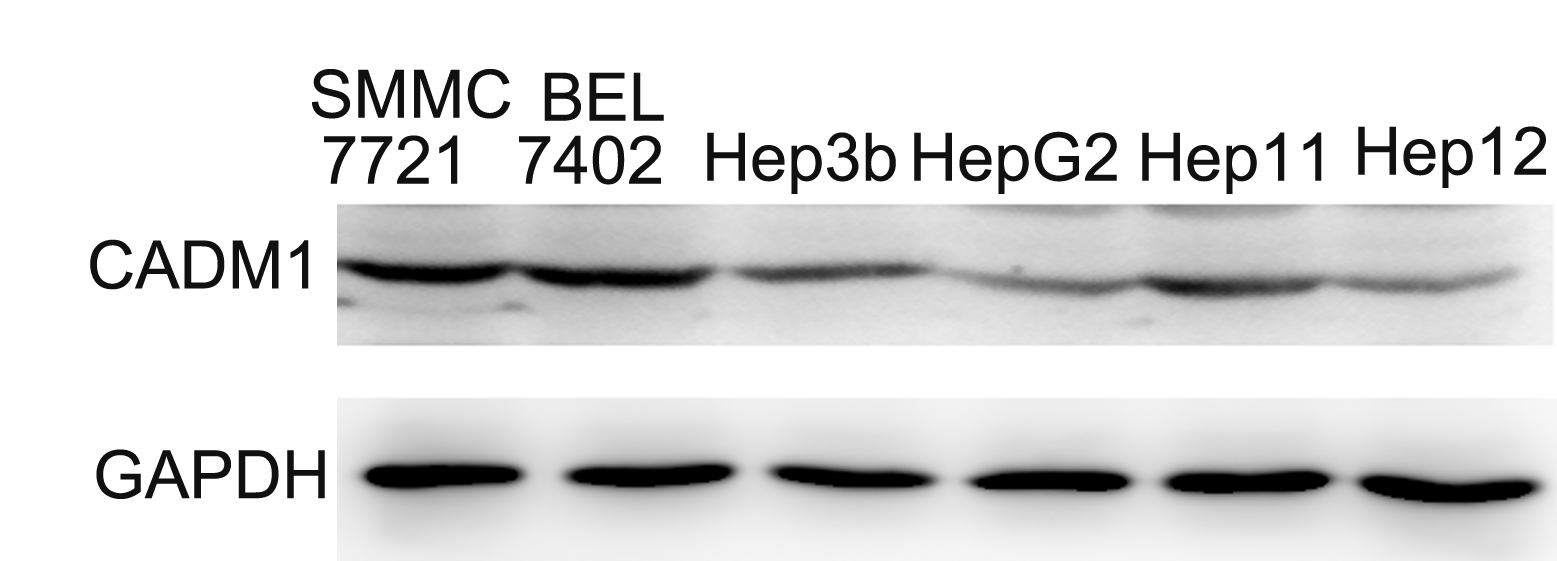

Supplement: Supplementary file 10 — Additional file 10: Figure S9: Western blot assay of CADM1 expression in different HCC cell lines. (TIFF 913 KB) [file 12885_2014_4790_MOESM10_ESM.tiff]

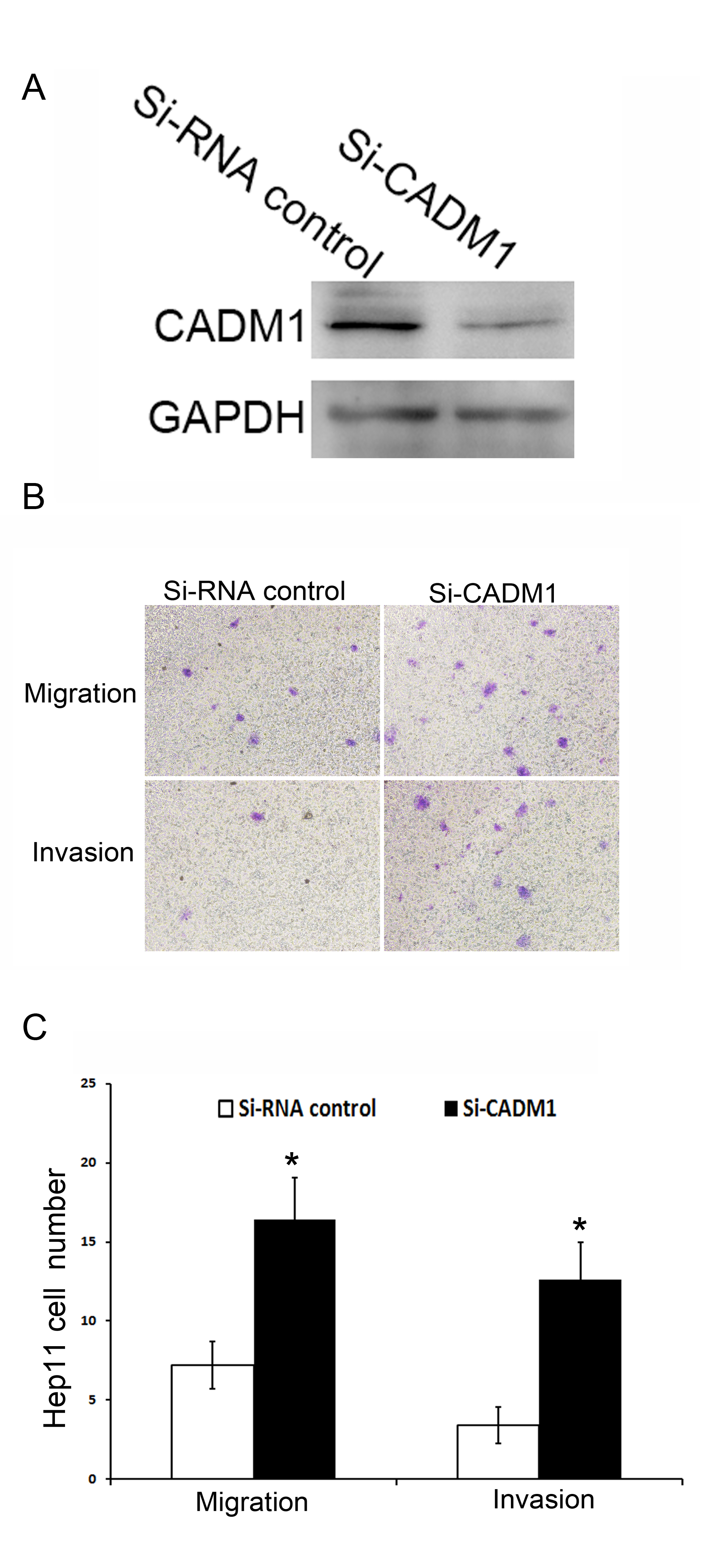

Supplement: Supplementary file 11 — Additional file 11: Figure S10: CADM1 knockdown in Hep11 promote migration and invasion. (A) Western blot assay showed that CADM1 were downregulated in Hep11 cells transfected with the CADM1 siRNA. (B, C) Transwell migration (n=4) and invasion (n=4) assays showed that Hep11 cells transfected with the CADM1 siRNA had greater invasive and migratory potentials than the control (siRNA control). (B) is a microscopic image of crystal violet staining; (C) shows the statistical results. (TIFF 4 MB) [file 12885_2014_4790_MOESM11_ESM.tiff]
